# Prediction of gastrointestinal hemorrhage in cardiology inpatients using an interpretable XGBoost model

**DOI:** 10.1038/s41598-025-10906-1

**Published:** 2025-07-12

**Authors:** Yahui Li, Xujie Wang, Xuhui Liu

**Affiliations:** 1 Division of Cardiology, Department of Internal Medicine, Tongji Hospital, Tongji Medical College, Hubei Key Laboratory of Genetics and Molecular Mechanisms of Cardiological Disorders, Huazhong University of Science and Technology, 1095 Jiefang Ave, Wuhan, 430030 Hubei China; 2 Department of Emergency ICU, The Affiliated Hospital of Qinghai University, Xining, China; 3 Department of Neurology, The Second Hospital of Lanzhou University, 82 Cuiyingmen, Chengguan District, Lanzhou, 730030 Gansu China; 4 Department of Neurology, The Second Hospital of Lanzhou University, 82 Cuiyingmen, Chengguan District, Lanzhou, 730030 Gansu China

**Keywords:** Gastrointestinal hemorrhage, Cardiovascular disease, Interpretability, Machine learning, XGBoost, Cardiovascular biology, Cardiology, Medical research

## Abstract

Gastrointestinal bleeding (GIB) occurs more frequently in cardiovascular patients than in the general population, significantly affecting morbidity and mortality. However, existing predictive models often lack sufficient accuracy and interpretability. We developed an interpretable and practical machine learning model to predict the risk of GIB in cardiology inpatients. This retrospective study analyzed electronic health records of 10,706 patients admitted to the Department of Cardiology at the Second Hospital of Lanzhou University from October 8, 2019, to October 30, 2024. Variables with > 30% missing data were excluded, leaving 35 potential predictors. The dataset was randomly split into a training cohort (80%, *n* = 9,356) and a test cohort (20%, *n* = 2,340). GIB occurred in 110 patients (1.03%). Ten variables were identified as the strongest predictors: hemoglobin (importance score: 0.16), creatinine (0.12), D-dimer (0.10), NT-proBNP (0.06), glucose (0.06), white blood cell count (0.06), body weight (0.06), serum albumin (0.04), urea (0.04), and age (0.04). Among seven machine learning classifiers, XGBoost performed best, with an AUC of 0.995 in the validation cohort. In the validation set, the model achieved an accuracy of 0.975, sensitivity of 0.769, and specificity of 0.996. SHapley Additive exPlanations (SHAP) analysis confirmed hemoglobin, creatinine, and D-dimer as the top contributors to GIB risk. The model demonstrated excellent calibration (Brier score = 0.016), and decision curve analysis supported its clinical utility across various risk thresholds. The XGBoost model offers high accuracy and interpretability in predicting GIB risk among cardiology inpatients. It holds promise for clinical decision support by enabling early risk identification and personalized prevention strategies.

**Supplementary Information:**

The online version contains supplementary material available at 10.1038/s41598-025-10906-1.

## Introduction

Gastrointestinal bleeding (GIB) is a serious complication in cardiology patients, associated with increased morbidity, mortality, and healthcare costs^1^. These patients are particularly vulnerable due to multiple factors, including widespread use of antithrombotic therapy, prevalent comorbidities, and impaired intestinal perfusion in conditions such as heart failure^2,3^. his heightened vulnerability highlights the urgent need for accurate risk prediction and effective management strategies. The incidence of GIB in cardiology patients is significantly higher than in the general population, with studies reporting rates as high as 5–10% among those receiving dual antiplatelet therapy or anticoagulation^4^. This risk is especially elevated in patients with acute coronary syndromes, atrial fibrillation, and heart failure^5^. Although antithrombotic agents are essential for preventing thromboembolic events, they also substantially increase GIB risk. For example, combining aspirin with a P2Y12 inhibitor can raise the risk of GIB two- to three-fold compared to aspirin alone^6^.

The delicate balance between preventing thrombotic events and minimizing bleeding risk poses a significant clinical challenge in the management of cardiology patients. Several factors have been identified as predictors of GIB in this population. Anemia, often resulting from occult bleeding or nutritional deficiencies, serves both as a marker and an independent risk factor for GIB^7^. The relationship is bidirectional—anemia may exacerbate cardiac ischemia, prompting increased use of antithrombotic agents, thereby further elevating bleeding risk. Renal dysfunction, prevalent in cardiovascular disease, contributes to platelet dysfunction and impaired drug metabolism, substantially increasing bleeding risk^8^. This is particularly concerning in patients requiring anticoagulation, such as those with atrial fibrillation, where careful dose adjustment and monitoring are essential. Elevated cardiac biomarkers like NT-proBNP may indicate severe cardiac dysfunction and splanchnic hypoperfusion, which can predispose the gastrointestinal mucosa to injury and bleeding^9^. This underscores the complex interplay between cardiac function and gastrointestinal health, highlighting the need for holistic patient management. Other notable predictors include advanced age-associated with vascular fragility, polypharmacy, and altered pharmacokinetics-and comorbidities such as diabetes mellitus, which impairs gastric mucosal defenses^10^. Inflammatory markers, such as elevated white blood cell count and C-reactive protein, have also been linked to increased GIB risk, possibly reflecting systemic inflammation that compromises mucosal integrity^11^. This intricate network of interrelated factors poses a challenge for clinicians using traditional risk prediction models. These models often rely on a limited set of variables and assume linear relationships, which may not adequately capture the complex, dynamic nature of risk in real-world patients^12^. In this context, machine learning (ML) offers distinct advantages in identifying nonlinear interactions and high-dimensional patterns in clinical data.

ML algorithms can process large volumes of complex, multidimensional data and uncover subtle patterns often missed by traditional statistical methods^13^. This capacity is particularly advantageous in predicting GIB risk, where numerous interdependent factors influence patient outcomes. In cardiovascular medicine, ML has already shown promise in forecasting outcomes such as mortality, hospital readmission, and major adverse cardiac events^14^. One major barrier to the clinical adoption of ML has been its “black box” nature. However, recent advancements in explainable artificial intelligence (XAI) have improved model transparency. Techniques such as SHAP (SHapley Additive exPlanations) provide interpretable insights into how individual variables contribute to specific predictions, thereby enhancing clinician understanding and confidence in ML-based tools^15^. This interpretability not only supports clinical decision-making but also helps generate new hypotheses regarding disease mechanisms.

Despite these advantages, the application of ML for GIB risk prediction in cardiology patients remains limited. Most existing studies rely on traditional statistical approaches or suffer from small sample sizes and narrow variable selection^16,17^. There is a clear need for comprehensive ML-based models capable of accurately stratifying GIB risk in this high-risk population. Such models may also reveal novel risk factors and complex interactions that have not been previously identified.

In this study, we aimed to develop and validate a machine learning model to predict the risk of gastrointestinal hemorrhage in cardiology inpatients. Utilizing a large dataset and advanced ML techniques, our goal was to establish a robust, accurate, and interpretable risk prediction tool. Such a model could facilitate personalized risk assessment and guide clinical decision-making, ultimately improving outcomes for this vulnerable patient population. Additionally, insights derived from this ML-based approach may inform future research and deepen our understanding of the pathophysiology of GIB in cardiology patients.

## Method

### Study population and data source

**Fig. 1:**
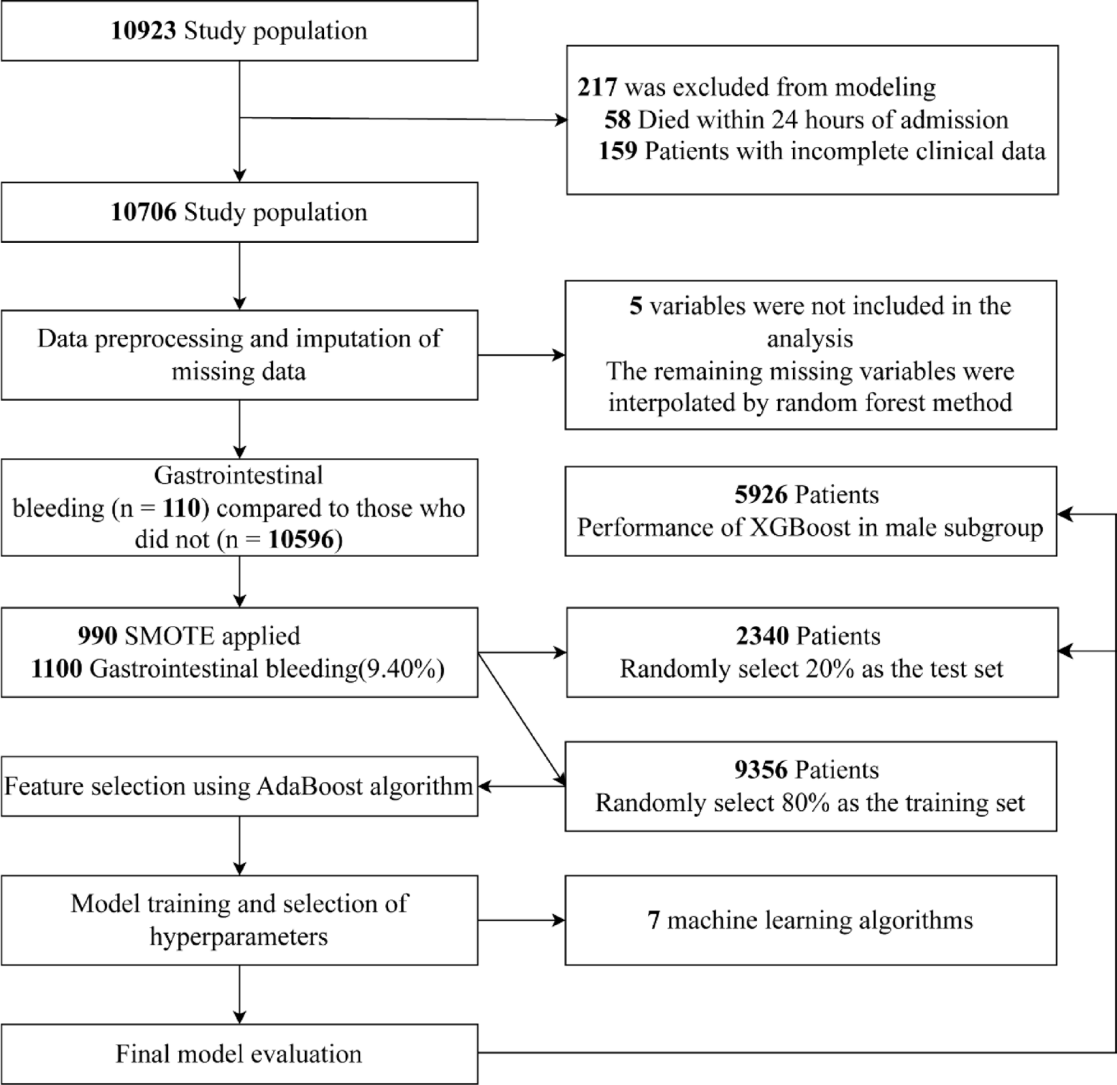
Flow chart of this study.

We retrospectively analyzed electronic medical records of 10,706 patients admitted to the Department of Cardiology at the Second Hospital of Lanzhou University from October 8, 2019, to October 30, 2024. Inclusion criteria were: (1) cardiology inpatients aged over 18 years; (2) hospital stay longer than 3 days; and (3) no diagnosis of gastrointestinal hemorrhage upon admission. Exclusion criteria included: (1) death within 24 h of admission; and (2) incomplete clinical data. The patient selection process is illustrated in Fig. 1. Ethical approval and consent to participate the retrospective design of the study received approval from the Ethics Committee of the Second Hospital of Lanzhou University (approval no. 2024 A-1271). The procedures used in this study adhere to the tenets of the Declaration of Helsinki. As this was a retrospective observational study, informed consent was waived by the Ethics Committee of the Second Hospital of Lanzhou University. The development and validation of the prediction model adhered to the Transparent Reporting of a Multivariable Prediction Model for Individual Prognosis or Diagnosis (TRIPOD) guidelines.

### Definition of gastrointestinal hemorrhage

GIB was defined based on international consensus^18^ and American Gastroenterological Association guidelines^19^. Diagnosis required meeting at least one of the following criteria: (1) hematemesis, melena, or hematochezia; (2) hemoglobin decrease ≥ 2 g/dL; (3) need for blood transfusion; or (4) endoscopically confirmed upper or lower Gastrointestinal hemorrhage.

### Feature selection and data preprocessing

The initial dataset included 40 variables. To ensure data quality and model robustness, we implemented a rigorous preprocessing strategy. Variables with more than 30% missing data were excluded: Glu_2h (89.99%), HbA1c (56.06%), renal anemia (47.54%), serum albumin (47.70%), and PO2 (48.80%). Remaining missing values were imputed using the random forest method. After preprocessing, 35 variables were retained for analysis. The dataset was randomly split into a training cohort (*n* = 9,356, 80%) and a test cohort (*n* = 2,340, 20%). The training cohort was used for model development and performance comparison, while the test cohort served for independent validation of the final model.

To mitigate multicollinearity, we performed collinearity analysis and retained variables with variance inflation factors (VIF) below 5. AdaBoost, which improves classification by sequentially training weak classifiers (typically decision trees), assigns higher weights to misclassified samples in each iteration. This adaptive weighting allows AdaBoost to focus on more informative features during feature selection. By analyzing feature importance within the AdaBoost classifier, we identified the top ten features that contributed most to the model’s predictive performance^20^.

### Machine learning model development and comparison

Seven machine learning algorithms were evaluated: XGBoost, Logistic Regression, LightGBM, Random Forest, Gradient Boosting Decision Tree (GBDT), Gaussian Naive Bayes (GNB) and K-Nearest Neighbors (KNN). Model performance was assessed using the area under the receiver operating characteristic curve (AUC), accuracy, specificity, sensitivity, F1 score, positive predictive value (PPV), and negative predictive value (NPV). AUC comparisons were conducted using DeLong’s test. The optimal cutoff value was determined based on the maximum Youden index.

### Optimal model performance evaluation

The best-performing classifier, XGBoost, was selected for final model development. The model was trained and validated using 10-fold cross-validation on the training cohort, repeated 10 times. Hyperparameters were manually tuned via grid search and calibrated on the validation set. Model performance was assessed based on discrimination metrics (AUC, accuracy, sensitivity, specificity, PPV, NPV, and F1 score), calibration (Brier score and calibration plots), and clinical utility (decision curve analysis). In our study, the Brier score was 0.016 in test set, indicating excellent agreement between predicted probabilities and observed outcomes.

### Model interpretability

To enhance clinical interpretability, we employed the SHapley Additive exPlanations (SHAP) algorithm, which utilizes a game-theoretic approach to explain the model’s predictions and highlight the contributions of key variables. SHAP analysis was conducted using the “SHAP” package version 0.39.0 on the validation cohort.

### Statistical analysis

All statistical analyses were performed using R version 4.3 (R Foundation for Statistical Computing) and Python version 3.7 (https://www.python.org/). Continuous variables were presented as median (interquartile range) or mean ± standard deviation and compared using the Mann-Whitney U test. Categorical variables were compared using the chi-square test or Fisher’s exact test. A p-value < 0.05 was considered statistically significant.

## Results

### Data preprocessing and feature selection

**Fig. 2:**
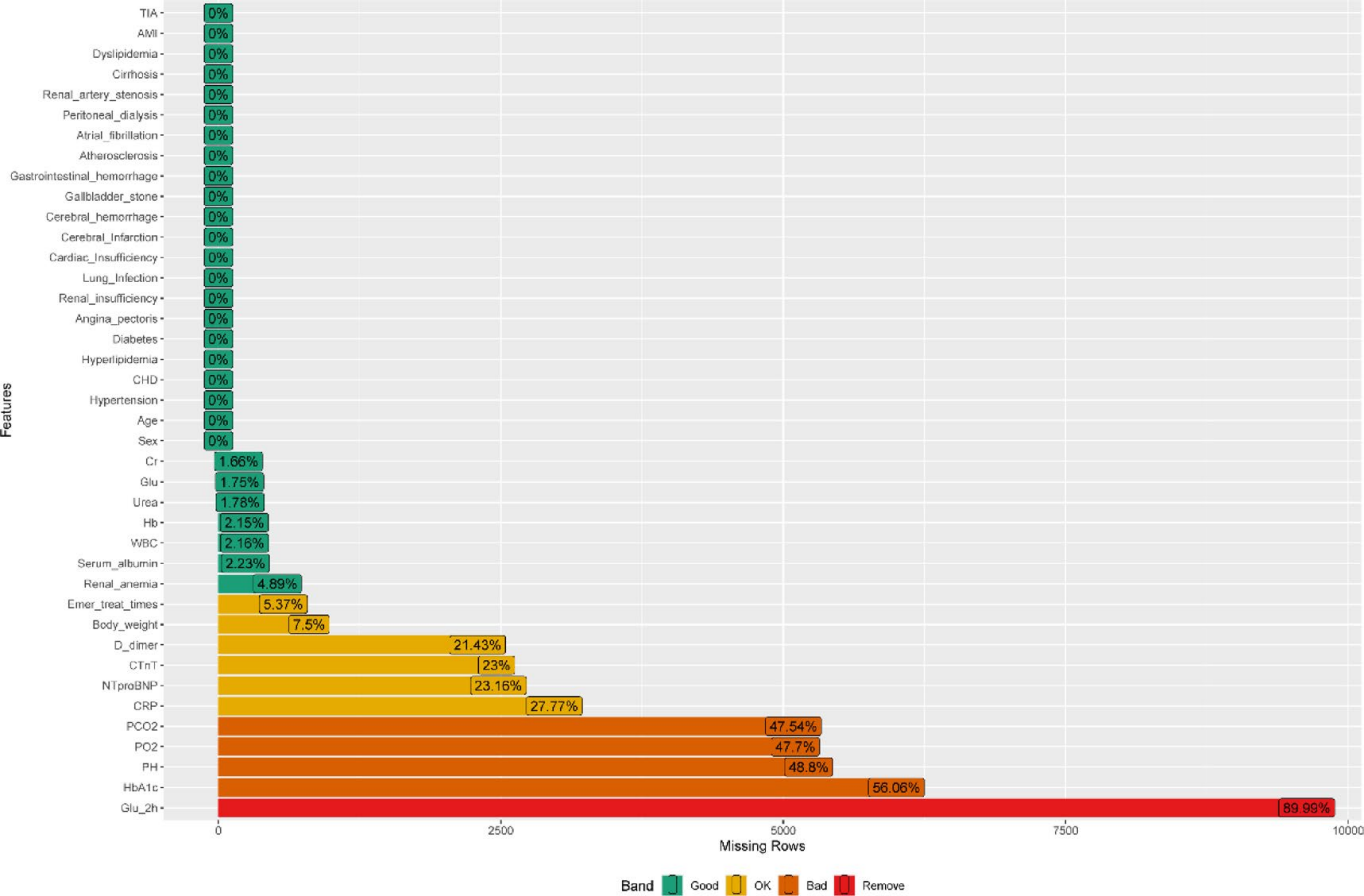
Bar plot showing the percentage of missing values for each variable. Variables are ordered by the percentage of missing data, with those exceeding 30% highlighted for exclusion.

We initially collected data on 40 variables from patients admitted to the cardiology department. To ensure data quality and model robustness, we implemented a rigorous preprocessing strategy. Figure 1 illustrates the percentage of missing values for each variable in our dataset. Variables with more than 30% missing data were excluded from further analysis to maintain model integrity. This criterion led to the removal of five variables: Glu_2h (89.99% missing), HbA1c (56.06%), renal anemia (47.54%), serum albumin (47.70%), and PO2 (48.80%) (Fig. 2). For the remaining variables, missing data were imputed using the random forest method, a sophisticated approach that preserves relationships among variables while providing accurate estimates for missing values. This method was selected for its ability to handle mixed data types and capture complex interactions, which is crucial in clinical datasets. After preprocessing, 35 variables were retained for subsequent analysis. These included demographic factors (sex, age), clinical measurements (body weight, CRP, WBC, cTnT, creatinine, NT-proBNP, urea, glucose, hemoglobin, D-dimer), blood gas parameters (PCO2, pH), and various comorbidities (hypertension, coronary heart disease, hyperlipidemia, diabetes, angina pectoris, renal insufficiency, lung infection, cardiac insufficiency, cerebral infarction, cerebral hemorrhage, gallbladder stone, gastrointestinal hemorrhage, atherosclerosis, atrial fibrillation, peritoneal dialysis, renal artery stenosis, cirrhosis, dyslipidemia, acute myocardial infarction, transient ischemic attack).

### Baseline characteristics of the study population

Our study included a total of 10,706 patients admitted to the cardiology department. We analyzed the characteristics of patients who experienced gastrointestinal hemorrhage (*n* = 110) compared to those who did not (*n* = 10,596). Specifically, we used the SMOTE method to synthetically increase the number of positive cases (i.e., gastrointestinal hemorrhage events) from the original 110 samples to 1,100 synthetic samples. The percentage of positive events was adjusted from 110/10,706 to 1,100/11,696. Subsequently, the cohort was randomly divided into a training set (*n* = 9,356) and a test set (*n* = 2,340) at a 8:2 ratio.

Table 1 presents the baseline characteristics of patients with and without gastrointestinal hemorrhage (left column) and the baseline characteristics of the training and test sets after SMOTE balancing (right column).

**Table 1:** Baseline characteristics of patients with and without Gastrointestinal hemorrhage (left column), and baseline characteristics of the training and test sets after SMOTE balancing (right column).

Variables	Total (*n* = 10706)	Gastrointestinal hemorrhage		*P*	Total (*n* = 11696)	Gastrointestinal hemorrhage		*P*
		No (*n* = 10596)	Yes (*n* = 110)			Training set (*n* = 9356)	Test set (*n* = 2340)	
Age, Mean ± SD	60.00 ± 14.92	59.88 ± 14.88	72.01 ± 14.24	< 0.001	61.06 ± 15.10	61.01 ± 15.10	61.25 ± 15.11	0.49
Emer treat times, Mean ± SD	0.05 ± 0.35	0.04 ± 0.34	0.32 ± 0.93	0.002	0.07 ± 0.42	0.07 ± 0.42	0.07 ± 0.40	0.622
Body weight, Mean ± SD	71.07 ± 13.39	71.11 ± 13.39	66.60 ± 12.49	< 0.001	70.64 ± 13.18	70.71 ± 13.21	70.37 ± 13.05	0.268
CRP, Mean ± SD	1.15 ± 4.51	1.13 ± 4.52	2.86 ± 3.69	< 0.001	1.30 ± 4.44	1.28 ± 4.67	1.37 ± 3.37	0.38
WBC, Mean ± SD	6.94 ± 2.79	6.93 ± 2.75	8.14 ± 5.37	0.019	7.04 ± 2.99	7.05 ± 2.99	7.02 ± 2.98	0.71
CTnT, Mean ± SD	0.05 ± 0.92	0.05 ± 0.93	0.09 ± 0.22	0.672	0.06 ± 0.88	0.06 ± 0.98	0.04 ± 0.31	0.223
Cr, Mean ± SD	97.63 ± 115.05	97.48 ± 115.15	112.11 ± 104.58	0.185	98.90 ± 113.41	98.91 ± 114.79	98.87 ± 107.74	0.989
NT-proBNP, Mean ± SD	977.05 ± 3962.02	936.60 ± 3835.61	4873.24 ± 9806.96	< 0.001	1299.64 ± 4798.69	1300.04 ± 4842.31	1298.01 ± 4621.20	0.985
Urea, Mean ± SD	6.42 ± 4.78	6.39 ± 4.72	9.32 ± 8.04	< 0.001	6.68 ± 5.14	6.67 ± 5.16	6.73 ± 5.05	0.599
Glu, Mean ± SD	6.31 ± 2.70	6.28 ± 2.68	8.39 ± 3.73	< 0.001	6.47 ± 2.78	6.48 ± 2.78	6.42 ± 2.81	0.302
Hb, Mean ± SD	129.91 ± 22.27	130.30 ± 21.96	92.48 ± 20.75	< 0.001	126.68 ± 24.38	126.65 ± 24.43	126.77 ± 24.20	0.825
D dimer, Mean ± SD	1.12 ± 2.35	1.09 ± 2.31	3.44 ± 3.83	< 0.001	1.32 ± 2.53	1.32 ± 2.54	1.30 ± 2.49	0.77
Serum albumin, Mean ± SD	40.14 ± 5.04	40.21 ± 5.00	33.70 ± 5.16	< 0.001	39.59 ± 5.31	39.59 ± 5.35	39.59 ± 5.14	0.974
Sex, n(%)				0.056				0.768
Female	5154 (48.14)	5111 (48.24)	43 (39.09)		5770 (49.33)	4622 (49.40)	1148 (49.06)	
Male	5552 (51.86)	5485 (51.76)	67 (60.91)		5926 (50.67)	4734 (50.60)	1192 (50.94)	
Hypertension, n(%)				0.049				0.109
No	366 (3.42)	358 (3.38)	8 (7.27)		509 (4.35)	393 (4.20)	116 (4.96)	
Yes	10,340 (96.58)	10,238 (96.62)	102 (92.73)		11,187 (95.65)	8963 (95.80)	2224 (95.04)	
CHD, n(%)				0.013				0.934
No	8763 (81.85)	8663 (81.76)	100 (90.91)		9745 (83.32)	7794 (83.30)	1951 (83.38)	
Yes	1943 (18.15)	1933 (18.24)	10 (9.09)		1951 (16.68)	1562 (16.70)	389 (16.62)	
Hyperlipidemia, n(%)				0.002				0.975
No	9868 (92.17)	9758 (92.09)	110 (100.00)		10,858 (92.84)	8686 (92.84)	2172 (92.82)	
Yes	838 (7.83)	838 (7.91)	0 (0.00)		838 (7.16)	670 (7.16)	168 (7.18)	
Diabetes, n(%)				0.691				0.611
No	8438 (78.82)	8353 (78.83)	85 (77.27)		9383 (80.22)	7497 (80.13)	1886 (80.60)	
Yes	2268 (21.18)	2243 (21.17)	25 (22.73)		2313 (19.78)	1859 (19.87)	454 (19.40)	
Anginta pectoris, n(%)				< 0.001				0.888
No	9411 (87.90)	9302 (87.79)	109 (99.09)		10,401 (88.93)	8322 (88.95)	2079 (88.85)	
Yes	1295 (12.10)	1294 (12.21)	1 (0.91)		1295 (11.07)	1034 (11.05)	261 (11.15)	
Rental insufficiency, n(%)				0.036				0.491
No	10,050 (93.87)	9952 (93.92)	98 (89.09)		11,001 (94.06)	8793 (93.98)	2208 (94.36)	
Yes	656 (6.13)	644 (6.08)	12 (10.91)		695 (5.94)	563 (6.02)	132 (5.64)	
Lung Infection, n(%)				0.046				0.039
No	10,469 (97.79)	10,365 (97.82)	104 (94.55)		11,459 (97.97)	9179 (98.11)	2280 (97.44)	
Yes	237 (2.21)	231 (2.18)	6 (5.45)		237 (2.03)	177 (1.89)	60 (2.56)	
Cardiac Insufficiency, n(%)				0.001				0.337
No	10,509 (98.16)	10,406 (98.21)	103 (93.64)		11,478 (98.14)	9176 (98.08)	2302 (98.38)	
Yes	197 (1.84)	190 (1.79)	7 (6.36)		218 (1.86)	180 (1.92)	38 (1.62)	
Cerebral Infarction, n(%)				< 0.001				0.833
No	10,435 (97.47)	10,334 (97.53)	101 (91.82)		11,423 (97.67)	9139 (97.68)	2284 (97.61)	
Yes	271 (2.53)	262 (2.47)	9 (8.18)		273 (2.33)	217 (2.32)	56 (2.39)	
Cerebral hemorrhage, n(%)				0.581				0.744
No	10,622 (99.22)	10,513 (99.22)	109 (99.09)		11,612 (99.28)	9290 (99.29)	2322 (99.23)	
Yes	84 (0.78)	83 (0.78)	1 (0.91)		84 (0.72)	66 (0.71)	18 (0.77)	
Gallbladder stone, n(%)				1				0.463
No	10,601 (99.02)	10,492 (99.02)	109 (99.09)		11,591 (99.10)	9275 (99.13)	2316 (98.97)	
Yes	105 (0.98)	104 (0.98)	1 (0.91)		105 (0.90)	81 (0.87)	24 (1.03)	
Atherosclerosis, n(%)				0.115				0.936
No	9383 (87.64)	9292 (87.69)	91 (82.73)		10,342 (88.42)	8274 (88.44)	2068 (88.38)	
Yes	1323 (12.36)	1304 (12.31)	19 (17.27)		1354 (11.58)	1082 (11.56)	272 (11.62)	
Atrial fibrillation, n(%)				0.842				0.391
No	10,491 (97.99)	10,384 (98.00)	107 (97.27)		11,481 (98.16)	9189 (98.22)	2292 (97.95)	
Yes	215 (2.01)	212 (2.00)	3 (2.73)		215 (1.84)	167 (1.78)	48 (2.05)	
Peritoneal dialysis, n(%)				1				0.693
No	10,696 (99.91)	10,586 (99.91)	110 (100.00)		11,686 (99.91)	9349 (99.93)	2337 (99.87)	
Yes	10 (0.09)	10 (0.09)	0 (0.00)		10 (0.09)	7 (0.07)	3 (0.13)	
Rental artery stenosis, n(%)				0.554				0.911
No	10,628 (99.27)	10,519 (99.27)	109 (99.09)		11,618 (99.33)	9294 (99.34)	2324 (99.32)	
Yes	78 (0.73)	77 (0.73)	1 (0.91)		78 (0.67)	62 (0.66)	16 (0.68)	
Cirrhosis, n(%)				< 0.001				0.729
No	10,641 (99.39)	10,538 (99.45)	103 (93.64)		11,620 (99.35)	9294 (99.34)	2326 (99.40)	
Yes	65 (0.61)	58 (0.55)	7 (6.36)		76 (0.65)	62 (0.66)	14 (0.60)	
AMI, n(%)				1				0.402
No	10,677 (99.73)	10,567 (99.73)	110 (100.00)		11,667 (99.75)	9331 (99.73)	2336 (99.83)	
Yes	29 (0.27)	29 (0.27)	0 (0.00)		29 (0.25)	25 (0.27)	4 (0.17)	
TIA, n(%)				1				0.168
No	10,668 (99.65)	10,558 (99.64)	110 (100.00)		11,658 (99.68)	9329 (99.71)	2329 (99.53)	
Yes	38 (0.35)	38 (0.36)	0 (0.00)		38 (0.32)	27 (0.29)	11 (0.47)	
Rental anemia, n(%)				1				0.683
No	10,582 (98.84)	10,473 (98.84)	109 (99.09)		11,572 (98.94)	9255 (98.92)	2317 (99.02)	
Yes	124 (1.16)	123 (1.16)	1 (0.91)		124 (1.06)	101 (1.08)	23 (0.98)	

### Comparison of patients with and without Gastrointestinal hemorrhage

Patients who experienced gastrointestinal hemorrhage were significantly older (72.01 ± 14.24 vs. 59.88 ± 14.88 years, *p* < 0.001) and had a higher number of emergency treatments (0.32 ± 0.93 vs. 0.04 ± 0.34, *p* = 0.002) compared to those without bleeding. They also exhibited significantly elevated inflammatory markers, including C-reactive protein (CRP: 2.86 ± 3.69 vs. 1.13 ± 4.52 mg/L, *p* < 0.001) and white blood cell count (WBC: 8.14 ± 5.37 vs. 6.93 ± 2.75 × 10^9/L, *p* = 0.019), as well as increased cardiac stress indicators (NT-proBNP: 4873.24 ± 9806.96 vs. 936.60 ± 3835.61 pg/mL, *p* < 0.001). Notably, patients with gastrointestinal hemorrhage had significantly lower hemoglobin levels (92.48 ± 20.75 vs. 130.30 ± 21.96 g/L, *p* < 0.001) and serum albumin levels (33.70 ± 5.16 vs. 40.21 ± 5.00 g/L, *p* < 0.001). They also showed elevated levels of D-dimer (3.44 ± 3.83 vs. 1.09 ± 2.31 mg/L, *p* < 0.001), urea (9.32 ± 8.04 vs. 6.39 ± 4.72 mmol/L, *p* < 0.001), and glucose (8.39 ± 3.73 vs. 6.28 ± 2.68 mmol/L, *p* < 0.001).

Regarding comorbidities, patients with gastrointestinal hemorrhage had a significantly higher prevalence of cirrhosis (6.36% vs. 0.55%, *p* < 0.001), cardiac insufficiency (6.36% vs. 1.79%, *p* = 0.001), and cerebral infarction (8.18% vs. 2.47%, *p* < 0.001). Conversely, they had a lower prevalence of hyperlipidemia (0% vs. 7.91%, *p* = 0.002) and angina pectoris (0.91% vs. 12.21%, *p* < 0.001) (Table 1).

### Comparison of training and validation sets after Smote

The cohort was divided into a training set (*n* = 9,356) and a test set (*n* = 2,340) at a 8:2 ratio. The two groups were well balanced, with no significant differences in most variables. Age (61.01 ± 15.10 vs. 61.25 ± 15.11 years, *p* = 0.49), sex (male: 50.60% vs. 50.94%, *p* = 0.768), and key clinical parameters—including CRP, WBC, NT-proBNP, and hemoglobin—were similar between the sets (all *p* > 0.05). The prevalence of comorbidities such as hypertension, coronary heart disease, diabetes, and renal insufficiency also showed no significant differences (all *p* > 0.05). The only exception was lung infection, which was slightly more frequent in the validation set (2.56% vs. 1.89%, *p* = 0.039); this minor difference is unlikely to affect model performance (Table 1).

### Feature selection using adaboost algorithm

**Fig. 3:**
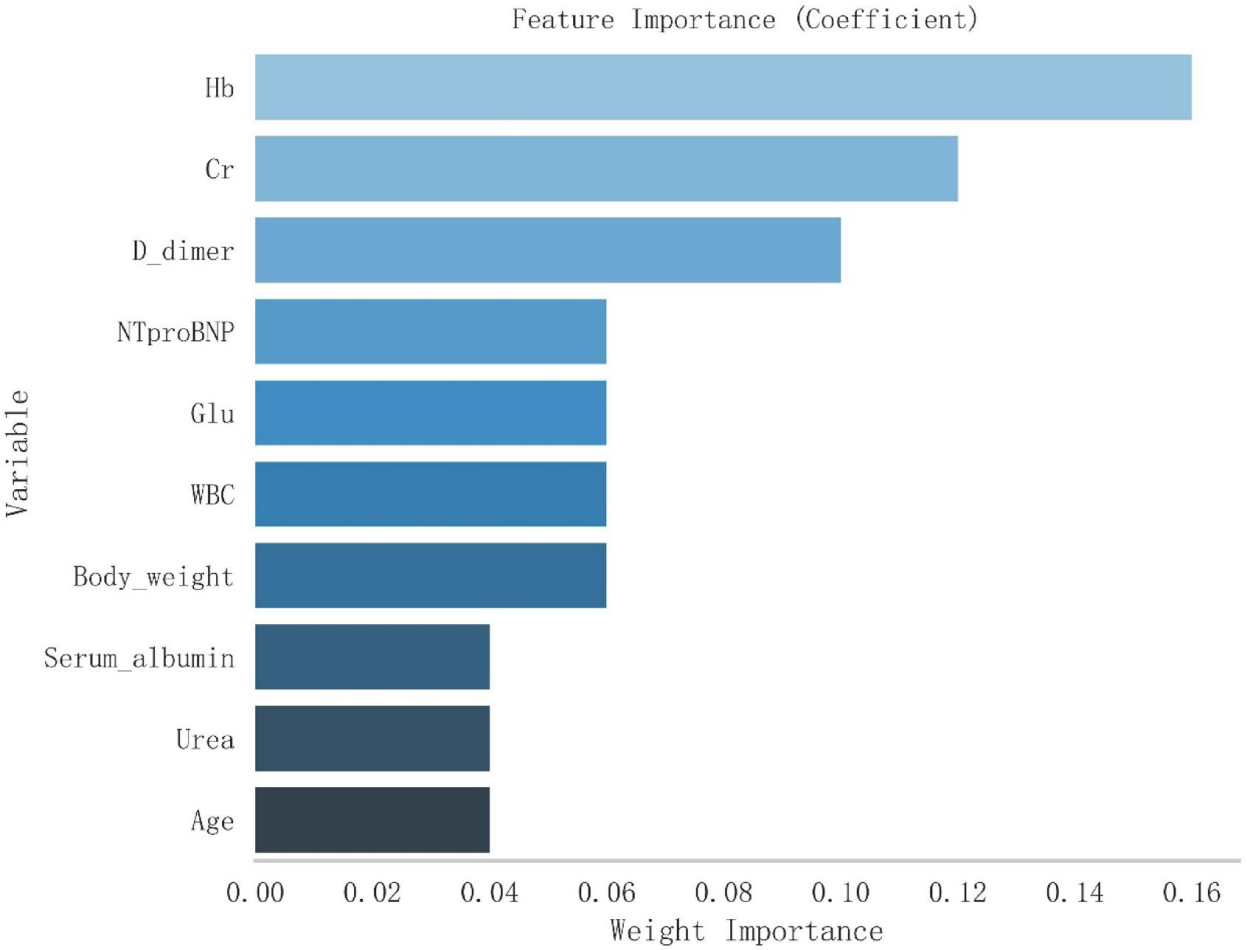
Feature Importance of Top 10 Variables Identified by AdaBoost Algorithm. The x-axis represents the weight importance, while the y-axis lists the variables. Longer bars indicate higher importance in the predictive model.

To identify the most influential predictors of gastrointestinal hemorrhage in cardiac patients, we applied the AdaBoost algorithm for feature selection. This approach ranked variables according to their contribution to the predictive model, allowing us to focus on the top 10 features for further analysis. Figure 3 displays the relative importance of these top 10 variables as horizontal bars, with bar length representing each variable’s weight in the model. Hemoglobin (Hb) was the most important predictor, followed by creatinine (Cr) and D-dimer. The remaining variables are shown in descending order of importance.

Table 2 presents the weight importance scores for these top 10 variables, providing a quantitative measure of their relative contribution to the predictive model. Hemoglobin demonstrated the highest importance score (0.16), followed by creatinine (0.12) and D-dimer (0.10). NT-proBNP, glucose, white blood cell count, and body weight each contributed equally with a score of 0.06. Serum albumin, urea, and age completed the top 10, each with an importance score of 0.04.

**Table 2:** Weight importance scores of top 10 variables from adaboost algorithm.

Variable	Weight Importance
Hb	0.16
Cr	0.12
D-dimer	0.10
NT-proBNP	0.06
Glu	0.06
WBC	0.06
Body weight	0.06
Serum albumin	0.04
Urea	0.04
Age	0.04

### Multicollinearity analysis of selected variables

**Fig. 4:**
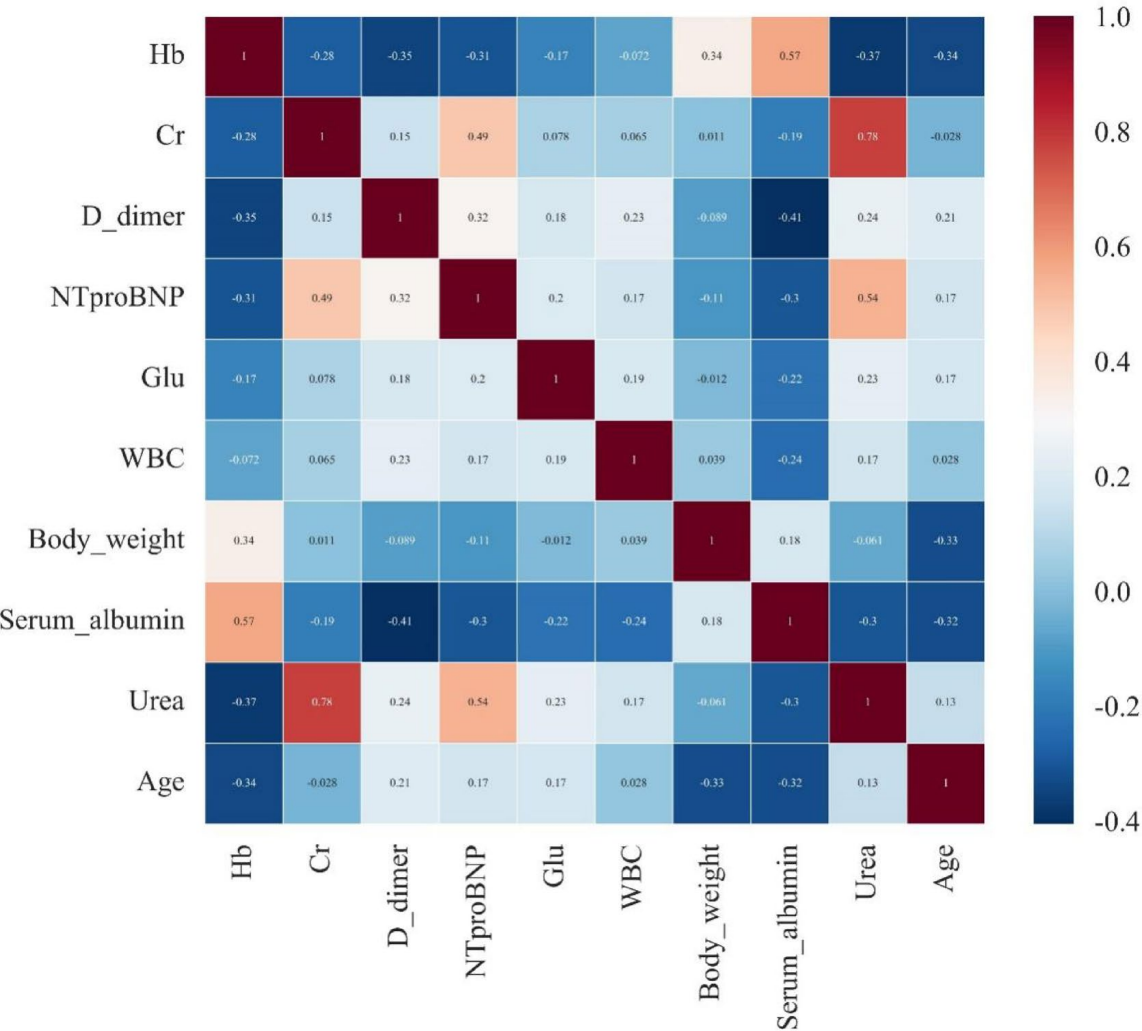
Correlation Matrix of the Top 10 Variables. The color intensity and size of the circles are proportional to the correlation coefficients. Blue circles indicate positive correlations, while red circles indicate negative correlations.

We conducted a multicollinearity analysis on the top 10 variables identified by the AdaBoost algorithm. Table 3 presents the results of variance inflation factor (VIF) calculations. The highest VIF value was 3.119 for urea, followed by 2.829 for creatinine. All other variables had VIF values below 2. Figure 4 displays the correlation matrix of the top 10 variables. The correlation matrix identified several key associations among variables: urea and creatinine demonstrated the strongest positive correlation (*r* = 0.78); hemoglobin positively correlated with serum albumin (*r* = 0.57) but negatively correlated with D-dimer (*r* = -0.35), NT-proBNP (*r* = -0.31), and urea (*r* = -0.37). Additionally, NT-proBNP showed moderate positive correlations with creatinine (*r* = 0.49) and urea (*r* = 0.54). Importantly, no correlation coefficient exceeded 0.8, indicating the absence of multicollinearity among the analyzed features.

**Table 3:** VIF for the top 10 variables.

Variable	VIF
Urea	3.119
Cr	2.829
Hb	1.824
Serum albumin	1.724
NT-proBNP	1.575
D-dimer	1.320
Age	1.306
Body weight	1.224
Glu	1.148
WBC	1.147

### Comparison of multiple machine learning models

**Fig. 5:**
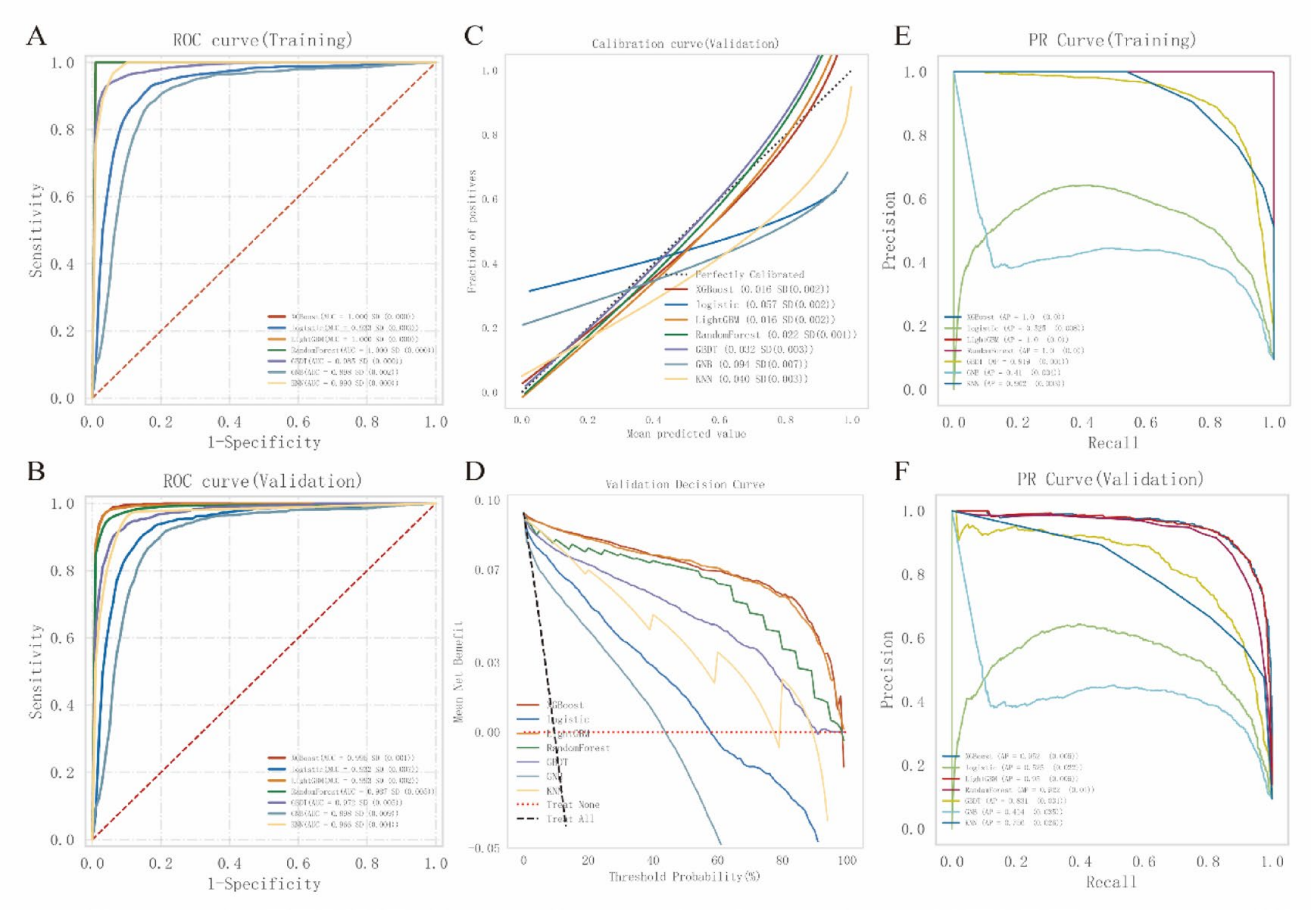
Performance comparison of machine learning models. (A) ROC curves for training set; (B) ROC curves for validation set; (C) Calibration curves for validation set; (D) Decision curve analysis for validation set; (E) PR curves for training set; (F) PR curves for validation set.

We evaluated seven machine learning models for their performance in predicting gastrointestinal hemorrhage in cardiac patients. Figure 5 provides a comprehensive comparison of these models across multiple performance metrics. Tables 4 and 5 summarize the performance metrics of all models in the training and validation sets, respectively.

**Table 4:** Performance metrics of machine learning models in the training set.

Classification Model	AUC(SD)	Cutoff(SD)	Accuracy(SD)	Sensitivity(SD)	Specificity(SD)	Positive predictive value(SD)	Negative predictive value(SD)	F1 Score(SD)	Kappa(SD)
XGBoost	1.000(0.000)	0.830(0.012)	1.000(0.000)	0.999(0.000)	1.000(0.000)	1.000(0.000)	1.000(0.000)	0.999(0.000)	0.999(0.000)
logistic	0.933(0.003)	0.082(0.004)	0.848(0.007)	0.919(0.007)	0.841(0.009)	0.377(0.012)	0.990(0.001)	0.534(0.011)	0.462(0.014)
LightGBM	1.000(0.000)	0.675(0.024)	1.000(0.000)	0.999(0.000)	1.000(0.000)	1.000(0.000)	1.000(0.000)	0.999(0.000)	0.999(0.000)
RandomForest	1.000(0.000)	0.470(0.024)	1.000(0.000)	0.997(0.001)	1.000(0.000)	0.999(0.001)	1.000(0.000)	0.998(0.001)	0.998(0.001)
GBDT	0.985(0.000)	0.200(0.048)	0.954(0.008)	0.933(0.008)	0.957(0.009)	0.695(0.043)	0.993(0.001)	0.796(0.026)	0.771(0.030)
GNB	0.898(0.002)	0.014(0.005)	0.834(0.009)	0.882(0.016)	0.829(0.012)	0.351(0.011)	0.985(0.002)	0.502(0.009)	0.424(0.012)
KNN	0.990(0.000)	0.400(0.000)	0.963(0.001)	0.887(0.008)	0.971(0.001)	0.765(0.006)	0.988(0.001)	0.821(0.006)	0.801(0.006)

**Table 5:** Performance metrics of machine learning models in the validation set.

Classification Model	AUC(SD)	Cutoff(SD)	Accuracy(SD)	Sensitivity(SD)	Specificity(SD)	Positive predictive value(SD)	Negative predictive value(SD)	F1 Score(SD)	Kappa(SD)
XGBoost	0.995(0.001)	0.830(0.012)	0.975(0.003)	0.769(0.044)	0.996(0.002)	0.959(0.020)	0.976(0.004)	0.852(0.023)	0.839(0.024)
logistic	0.932(0.007)	0.082(0.004)	0.847(0.006)	0.915(0.011)	0.840(0.006)	0.375(0.010)	0.990(0.001)	0.532(0.010)	0.459(0.012)
LightGBM	0.993(0.002)	0.675(0.024)	0.979(0.003)	0.841(0.046)	0.993(0.002)	0.928(0.018)	0.984(0.005)	0.881(0.021)	0.869(0.023)
RandomForest	0.987(0.005)	0.470(0.024)	0.976(0.002)	0.840(0.026)	0.990(0.004)	0.899(0.035)	0.983(0.003)	0.867(0.009)	0.854(0.011)
GBDT	0.972(0.005)	0.200(0.048)	0.942(0.013)	0.884(0.031)	0.948(0.017)	0.652(0.072)	0.987(0.003)	0.747(0.037)	0.715(0.043)
GNB	0.898(0.009)	0.014(0.005)	0.832(0.022)	0.880(0.018)	0.827(0.026)	0.350(0.029)	0.985(0.002)	0.500(0.030)	0.422(0.037)
KNN	0.966(0.004)	0.400(0.000)	0.943(0.004)	0.806(0.036)	0.958(0.004)	0.666(0.021)	0.979(0.004)	0.729(0.022)	0.697(0.024)

In the training set (Fig. 5A), XGBoost, LightGBM, and Random Forest all achieved perfect AUC scores of 1.000 (SD: 0.000). However, their performance varied in the validation set (Fig. 5B). XGBoost demonstrated the highest AUC at 0.995 (SD: 0.001), followed closely by LightGBM (AUC: 0.993, SD: 0.002) and Random Forest (AUC: 0.987, SD: 0.005). In terms of accuracy, XGBoost led with 0.975 (SD: 0.003), exhibiting a sensitivity of 0.769 (SD: 0.044) and specificity of 0.996 (SD: 0.002). LightGBM and Random Forest showed comparable accuracies of 0.979 (SD: 0.003) and 0.976 (SD: 0.002), respectively.

**Fig. 6:**
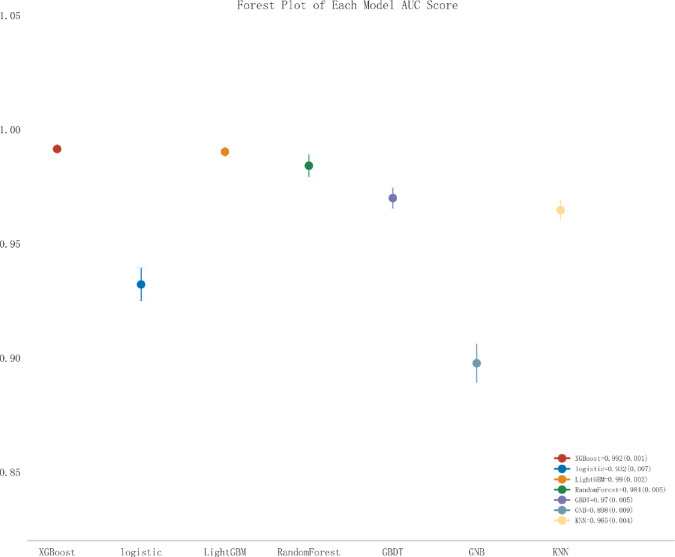
Multi-model forest plot for validation set.

Logistic Regression exhibited consistent but lower performance, achieving an AUC of 0.932 (SD: 0.007) in the validation set. It had the highest sensitivity (0.915, SD: 0.011) among all models but at the expense of lower specificity (0.840, SD: 0.006). Calibration curves (Fig. 5C) indicated that XGBoost, LightGBM, and Random Forest were well-calibrated, closely following the ideal line, whereas Gaussian Naive Bayes deviated notably. Decision curve analysis (Fig. 5D) further demonstrated the superior clinical utility of XGBoost, LightGBM, and Random Forest across a broad range of threshold probabilities (0.1–0.8), consistently yielding higher net benefits than “treat all” or “treat none” approaches. Precision-Recall curves (Fig. 5E and F) highlighted the robust performance of these three models in both training and validation sets, maintaining high precision over varying recall despite class imbalance. The forest plot (Fig. 6) visually confirmed their superior performance with non-overlapping confidence intervals compared to other models. DeLong’s test results (Supplementary Tables 1 and 2) showed that XGBoost significantly outperformed all models (*p* < 0.05), except for LightGBM (*p* = 0.379) and Random Forest (*p* = 0.035).

### XGBoost as the optimal predictive model

**Fig. 7:**
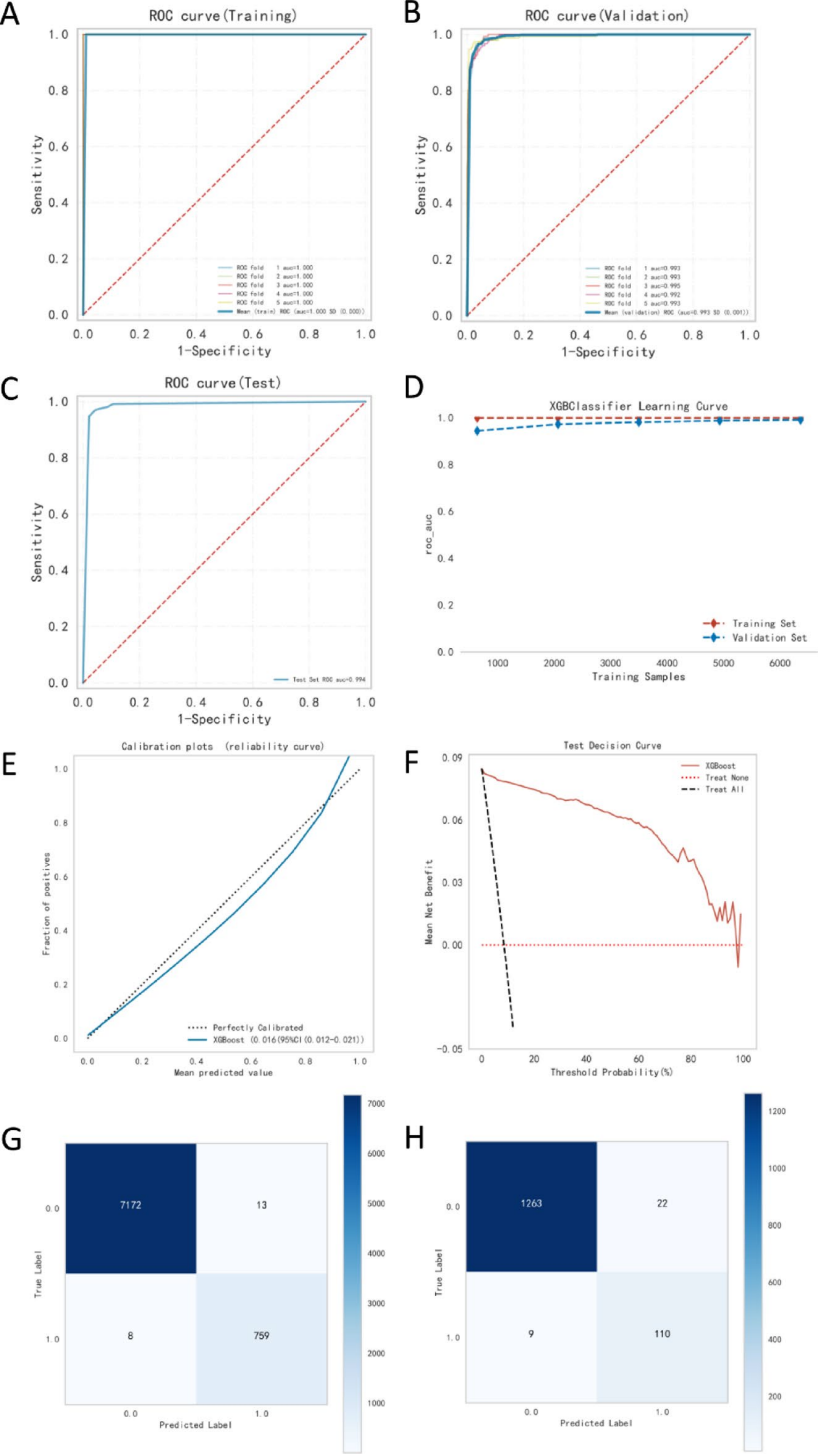
Performance evaluation of the XGBoost model. (A) ROC curve for the training set; (B) ROC curve for the validation set; (C) ROC curve for the test set; (D) Learning curve; (E) Decision curve; (F) Confusion matrix for the training set; (G) Confusion matrix for the validation set; (H) Calibration curve.

After a comprehensive evaluation of multiple machine learning algorithms, XGBoost was identified as the most effective model for predicting gastrointestinal hemorrhage in cardiac patients. Figure 7 illustrates its performance across various evaluation metrics. A detailed summary of the model’s performance in the training, validation, and test sets is provided in Table 6.

**Table 6:** Performance metrics of the XGBoost model in training, validation, and test sets.

Set	AUC	Cutoff	Accuracy	Sensitivity	Specificity	PPV	NPV	F1 Score
Training	1.000 (0.000)	0.840 (0.015)	1.000 (0.000)	0.999 (0.000)	1.000 (0.000)	1.000 (0.000)	1.000 (0.000)	0.999 (0.000)
Validation	0.994 (0.003)	0.840 (0.015)	0.973 (0.005)	0.767 (0.061)	0.995 (0.002)	0.949 (0.022)	0.976 (0.006)	0.846 (0.033)
Test	0.995	0.816	0.974	0.790	0.991	0.895	0.981	0.839

The XGBoost model demonstrated excellent discriminative performance across all datasets. In the training set, it achieved near-perfect results with an AUC of 1.000 (SD: 0.000) and an accuracy of 1.000 (SD: 0.000) (Fig. 7A; Table 6). This high performance was largely sustained in the validation set, with an AUC of 0.994 (SD: 0.003) and an accuracy of 0.973 (SD: 0.005) (Fig. 7B; Table 6). Importantly, the model maintained robust performance in the independent test set, reaching an AUC of 0.995 and an accuracy of 0.974 (Fig. 7C; Table 6). The comparative analysis in Supplementary Table 3 demonstrates that the XGBoost model significantly outperformed other algorithms in the independent test set: its AUC (0.995) was notably higher than AdaBoost (0.962) and Decision Tree (0.896). While maintaining high specificity (0.991), XGBoost achieved superior sensitivity (0.790) and F1-score (0.839) compared to Decision Tree (0.819 and 0.786, respectively). Although AdaBoost showed higher sensitivity (0.896), its specificity (0.901) and positive predictive value (0.513) were significantly lower. These results conclusively demonstrate XGBoost’s clear advantage in overall performance.

The learning curve (Fig. 7D) indicates that training and validation scores converged as sample size increased, suggesting effective learning and minimal overfitting. Decision curve analysis (Fig. 7E) showed that XGBoost provided consistently greater net benefit than the “treat all” or “treat none” strategies across a wide range of threshold probabilities (approximately 0.1 to 0.8), highlighting its clinical utility.

Confusion matrices (Fig. 7F–G) revealed strong classification performance. In the validation set, the model achieved high overall accuracy (0.973, SD: 0.005), with a sensitivity of 0.767 (SD: 0.061) and specificity of 0.995 (SD: 0.002). The calibration curve (Fig. 7H) closely aligned with the ideal diagonal, confirming good model calibration. In the test set, XGBoost continued to perform well, with a sensitivity of 0.790, specificity of 0.991, PPV of 0.895, and NPV of 0.981, reflecting excellent predictive reliability for both positive and negative cases.

The optimal cutoff value, determined by the Youden index, remained stable across datasets: 0.840 (SD: 0.015) in both training and validation sets, and 0.816 in the test set. This consistency further supports the model’s robustness.

### Superior performance of XGBoost in male subgroup

**Fig. 8:**
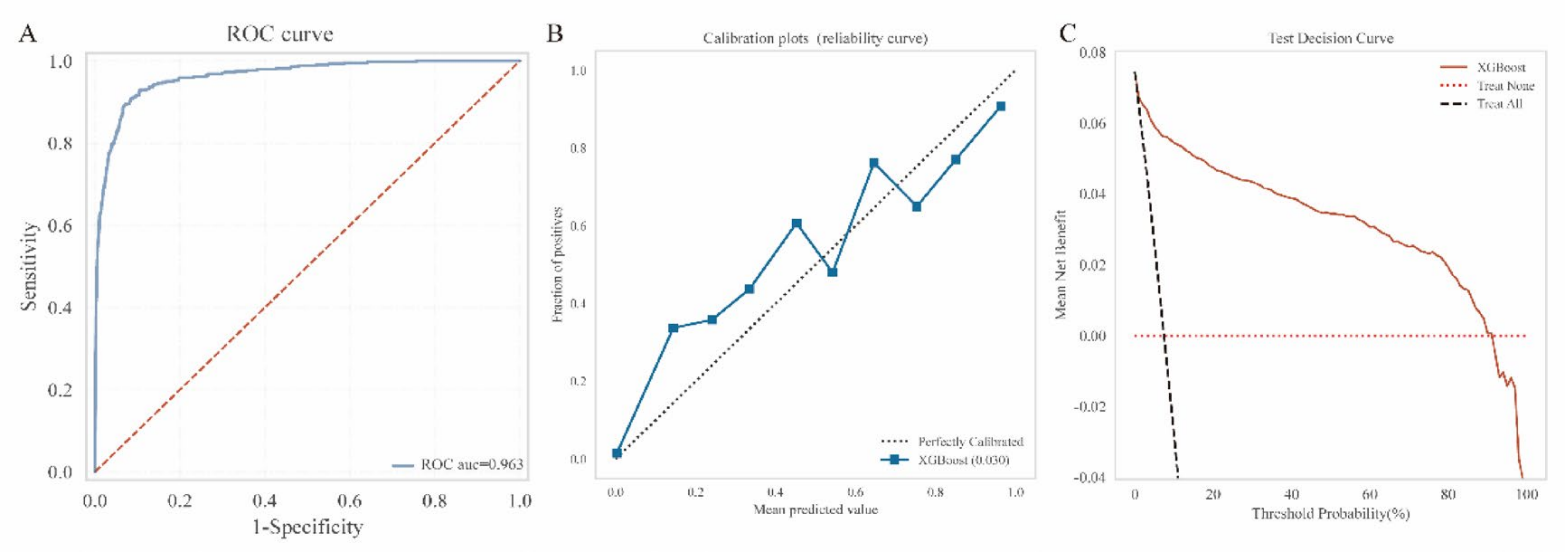
Performance evaluation of the XGBoost model in the male subgroup (*n* = 5,926). (A) ROC curve. (B) Calibration plot. (C) DCA curve.

The XGBoost model demonstrated robust predictive performance in the male subgroup. The ROC curve (Fig. 8A) exhibited excellent discriminative ability, with an AUC of 0.96, indicating strong capacity to distinguish between positive and negative cases. At the optimal cutoff threshold of 0.823, the model achieved a balanced sensitivity of 0.517 and a high specificity of 0.995, reflecting its precision in ruling out non-cases while maintaining moderate case detection. The calibration curve (Fig. 8B) showed close alignment between predicted probabilities and observed outcomes (Brier score = 0.030), suggesting reliable risk estimation across the spectrum of probabilities. Minor deviations at higher risk ranges may reflect limited sample size in extreme subgroups. DCA (Fig. 8C) further validated clinical utility, with the model providing net benefit over the “Treat All” and “Treat None” strategies across threshold probabilities of 10-80%. This supports its potential for guiding individualized decision-making in male patients.

### Interpretability and feature importance of the XGBoost model

**Fig. 9:**
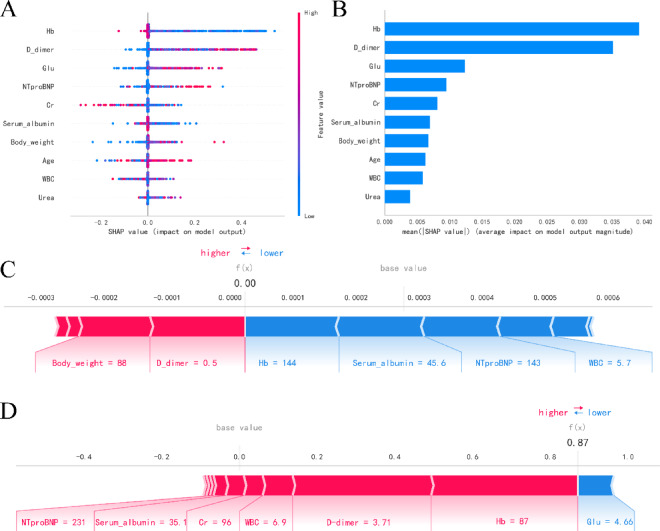
SHAP analysis of the XGBoost model. (A) SHAP summary plot; (B) Feature importance ranking; (C) SHAP force plot illustrating feature contributions for a representative negative prediction; (D) SHAP force plot illustrating feature contributions for a representative positive prediction.

To quantify the contribution of individual features to the predictions of the XGBoost model, we performed SHAP analysis. The results are illustrated in Fig. 9.

SHAP analysis of the XGBoost model (Fig. 9) revealed key feature contributions to predictions. The SHAP summary plot (Fig. 9A) demonstrated that hemoglobin (Hb) exhibited the strongest influence on model output (SHAP range: -0.2 to 0.4), where lower values (blue) were associated with increased risk and higher values (red) with decreased risk. D-dimer and NT-proBNP showed moderate positive correlations with risk (SHAP peaks ≈ 0.2), while other features like serum albumin and creatinine had minimal effects (|SHAP|<0.1). Feature importance ranking by mean absolute SHAP values (Fig. 9B) confirmed Hb as the most predictive feature (mean|SHAP|=0.035), followed by D-dimer (0.025) and NT-proBNP (0.015). Body weight, age, and urea showed negligible contributions (all < 0.01). Individual prediction analysis (Figs. 8C-D) illustrated these mechanisms: A negative case (Fig. 9C) showed protective effects from high Hb (144) and serum albumin (45.6), counterbalancing mild risk factors to maintain prediction near baseline (-0.0001). Conversely, a positive case (Fig. 9D) demonstrated how extreme risk factors - critically low Hb (87), elevated D-dimer (3.71), and high NT-proBNP (231) - drove the prediction to 0.6 despite some protective factors.

## Discussion

### Overview of key findings

This study aimed to develop and validate a machine learning model to predict the risk of GIB in cardiology inpatients. Our results indicate that the XGBoost model outperformed all other machine learning algorithms, achieving an AUC of 0.995 in the test set. Its high predictive accuracy, along with excellent calibration and clinical utility, suggests that the proposed model could serve as a reliable tool for early risk stratification and prevention of GIB among hospitalized cardiac patients^21^.

### High risk of Gastrointestinal hemorrhage in cardiology patients and its mechanisms

The incidence of GIB in our study population was 1.03%, markedly higher than the reported rate in the general population (0.1–0.2%)^1^. This elevated risk among cardiology inpatients may be explained by several factors. First, cardiovascular diseases and GIB share common risk factors, including advanced age and multiple comorbidities^22^. Second, commonly prescribed antithrombotic therapies in cardiology significantly increase bleeding risk^2^. Antiplatelet agents (e.g., aspirin and P2Y₁₂ inhibitors) impair platelet aggregation and can aggravate mucosal injury in the gastrointestinal tract^6^. Anticoagulants (e.g., warfarin and non-vitamin K oral anticoagulants [NOACs]) are essential for preventing thromboembolism but are also associated with a heightened risk of gastrointestinal bleeding^23^. The concurrent use of antiplatelet and anticoagulant agents-often necessary in patients with both coronary artery disease and atrial fibrillation-further increases this risk^4^. Moreover, pathophysiological changes in heart failure can predispose patients to GIB. Reduced cardiac output may cause mesenteric ischemia, compromising the gastrointestinal mucosal barrier^24^. In addition, venous congestion may lead to portal hypertension and the development of gastroesophageal varices, a major source of upper GIB^25^.

Previous studies have reported GIB rates of 5–10% in high-risk populations, particularly among patients receiving dual antiplatelet therapy (DAPT) or anticoagulation^4^. However, our study population differs in several key aspects that may explain the lower overall incidence of 1.03%: Broader inclusion criteria: Our cohort included all cardiology inpatients, not only those on DAPT or anticoagulation. While many patients in our dataset received such therapies, not all did, thereby lowering the overall GIB rate. Preventive measures: In our institution, routine gastrointestinal prophylaxis (e.g., proton pump inhibitors) is administered to patients receiving antithrombotic therapy, which likely reduced the risk of GIB substantially. Exclusion of patients with prior GIB: Patients with recent or active GIB are often excluded from aggressive antithrombotic regimens or managed in non-cardiology departments, potentially reducing the observed incidence in our sample. Population characteristics: The study was conducted in a tertiary hospital with standardized care protocols and close inpatient monitoring, which may have facilitated early identification and management of GIB risk factors. Therefore, although the observed incidence appears lower than in some previously reported high-risk groups, we believe that the 1.03% rate accurately reflects the real-world burden of GIB among contemporary cardiology inpatients receiving individualized care and modern preventive strategies.

### Clinical significance of key predictors and their association with GIB mechanisms

Our model identified several key predictors of GIB risk in cardiology patients, each with distinct clinical implications and underlying pathophysiological mechanisms. Hb emerged as the most influential predictor. Low Hb levels may not only result from GIB but also serve as an independent risk factor. Anemia can exacerbate myocardial ischemia, often prompting the use of antithrombotic therapies and thereby increasing bleeding risk^26^. Moreover, anemia may indicate underlying comorbidities or nutritional deficiencies that predispose patients to GIB^27^. The pathophysiological link is multifactorial: iron deficiency can impair gastrointestinal mucosal regeneration, making the tissue more vulnerable to injury^28^ ; compensatory mechanisms such as increased cardiac output and tachycardia in anemia may further reduce gastrointestinal perfusion^29^.

Cr, indicative of renal function, was the second most important predictor. Impaired renal function increases bleeding risk through mechanisms such as platelet dysfunction, altered drug metabolism, and uremic gastritis^30^. Uremia leads to defects in platelet aggregation and adhesion, prolongs bleeding time, and disrupts interactions with the vascular endothelium^31^. Additionally, renal impairment often coexists with cardiovascular disease, necessitating complex medication management to balance thrombotic and bleeding risks^5^. Impaired renal clearance may also lead to drug accumulation and increased bleeding potential^32^.

D-dimer, a marker of fibrinolytic activity, was also a significant predictor. Elevated D-dimer levels may reflect a hypercoagulable state managed with intensified anticoagulation, thus increasing GIB risk^33^. This marker may signal subclinical bleeding or heightened thrombin activity, both of which are associated with gastrointestinal mucosal injury^34^. Furthermore, microvascular thrombosis from excessive fibrin turnover can result in ischemic mucosal damage and subsequent bleeding^35^.

NT-proBNP, a biomarker of cardiac stress and heart failure severity, demonstrated strong predictive value. Elevated NT-proBNP levels reflect worse cardiac function and are linked to splanchnic hypoperfusion and mucosal ischemia, both of which increase GIB susceptibility^3^. Higher levels also often necessitate aggressive anticoagulant therapy in patients with reduced ejection fraction, further compounding bleeding risk^36^. Blood glucose levels also contributed significantly. Hyperglycemia compromises gastric mucosal defenses and delays healing, thereby elevating the risk of GIB^37^. Mechanistically, oxidative stress, advanced glycation end products, and microvascular dysfunction all impair mucosal integrity^38^. Diabetes, commonly associated with hyperglycemia, is a known risk factor for peptic ulcers and related complications, including bleeding^10^.

WBC count was another important predictor. Elevated WBC counts may indicate systemic inflammation or infection, which can heighten GIB risk, especially in patients on antithrombotic therapy^39^. Inflammatory processes increase vascular permeability and promote mucosal erosions^40^. Additionally, inflammation disrupts the balance between prothrombotic and fibrinolytic systems, thereby exacerbating bleeding tendencies in patients receiving antithrombotics^11^.

### Advantages of the XGBoost model and its clinical application prospects

The XGBoost model demonstrated superior performance compared to other machine learning algorithms in our study. With a high AUC of 0.995, accuracy of 0.974, and a low Brier score of 0.016, the model exhibited strong discriminative ability, excellent calibration, and robust predictive reliability across a range of risk thresholds^13^.

A key advantage of the model lies in its interpretability, addressed through SHAP analysis^41^. SHAP values quantify the individual contribution of each feature to a specific prediction, thereby transforming the XGBoost model from a “black box” into a transparent decision-support tool. This transparency is critical for building clinician trust, facilitating clinical integration, and enabling informed, individualized decision-making.

The XGBoost model’s combination of high predictive performance and interpretability lends itself to several potential clinical applications. For instance, it could be embedded within electronic health record (EHR) systems to provide real-time, automated risk stratification for GIB among cardiology inpatients. Such functionality would allow for early identification of high-risk individuals and prompt implementation of preventive strategies or intensified monitoring^42^.

Furthermore, the model could aid in personalized treatment planning—particularly in optimizing antithrombotic therapy or timing of invasive procedures—by weighing the risk of thrombotic events against the likelihood of GIB^12^. Overall, the model offers a promising tool to enhance clinical decision-making and improve patient outcomes in a high-risk population.

### Innovative aspects of the study

This study represents a significant advancement in the application of machine learning to cardiovascular risk prediction. While prior research has leveraged machine learning for forecasting various cardiovascular outcomes^14^our study uniquely focuses on GIB among cardiology inpatients—an important but frequently under-recognized complication. By utilizing XGBoost, a powerful ensemble learning algorithm, we were able to capture complex, non-linear interactions among clinical features that traditional statistical models may fail to detect^43^. A key strength of our work lies in its comprehensive evaluation framework, which incorporated measures of discrimination, calibration, and clinical utility. Such a multi-dimensional assessment aligns with current best practices and recommendations for clinical prediction model validation^44^providing a robust foundation for potential clinical implementation.

Moreover, the integration of SHAP analysis adds an innovative dimension to our methodology. SHAP offers feature-level interpretability, thus transforming the model into a transparent decision-support tool rather than a “black box“^15^. This not only enhances user trust and clinical adoption but also yields mechanistic insights into the relative importance and directional influence of key predictors. Such interpretability can support informed decision-making and guide hypothesis generation for future clinical and translational research^45^.

### Study limitations and future directions

Despite its strengths, this study has several limitations. Being a single-center investigation, the generalizability of our findings to other populations or healthcare settings may be limited. Additionally, some potentially important variables, such as detailed medication regimens, were unavailable in our dataset. Our focus was restricted to inpatient GIB events, thus post-discharge bleeding episodes were not captured. Future research should aim to address these limitations through multi-center validation studies, incorporation of additional clinically relevant variables, and prospective real-time evaluation of the model’s performance in clinical practice. Further exploration of the model’s effectiveness in specific patient subgroups and its integration with existing risk assessment tools could also enhance its clinical applicability.

### Conclusion

In conclusion, our study highlights the potential of machine learning—particularly the XGBoost algorithm—in accurately predicting gastrointestinal bleeding risk among cardiology inpatients. The model’s high predictive accuracy, robust calibration, and enhanced interpretability via SHAP analysis mark a significant advancement in risk stratification for this critical clinical complication. By identifying high-risk patients, the model can support targeted preventive measures, optimize antithrombotic therapy, and inform clinical decision-making, ultimately contributing to reduced morbidity and mortality in this vulnerable population.

## Electronic supplementary material

Below is the link to the electronic supplementary material.

Supplementary Material 1

## Data Availability

Data availabilityThe raw data is available from the corresponding author (liuxuhui_1995@yeah.net) on reasonable request.

## References

[1] Palmer, R. H. Risk of upper and lower Gastrointestinal bleeding in patients taking nonsteroidal Anti-Inflammatory drugs, antiplatelet agents, or anticoagulants. *Clin. Gastroenterol. Hepatol.* **13**, 2023–2024 (2015). 26027546 10.1016/j.cgh.2015.05.025

[2] Cheung, K. S. & Leung, W. K. Gastrointestinal bleeding in patients on novel oral anticoagulants: risk, prevention and management. *World J. Gastroenterol.* **23**, 1954–1963 (2017). 28373761 10.3748/wjg.v23.i11.1954 PMC5360636

[3] Sundaram, V. & Fang, J. C. Gastrointestinal and liver issues in heart failure. *Circulation* **133**, 1696–1703 (2016). 27143152 10.1161/CIRCULATIONAHA.115.020894

[4] Hamon, M. et al. Consensus document on the radial approach in percutaneous cardiovascular interventions: position paper by the European association of percutaneous cardiovascular interventions and working groups on acute cardiac Care** and Thrombosis of the European Society of Cardiology. *EuroIntervention* **8**, 1242–1251 (2013). 23354100 10.4244/EIJV8I11A192

[5] Kumar, S. et al. Anticoagulation in concomitant chronic kidney disease and atrial fibrillation: JACC review topic of the week. *J. Am. Coll. Cardiol.* **74**, 2204–2215 (2019). 31648714 10.1016/j.jacc.2019.08.1031

[6] Scheiman, J. M. NSAID-induced Gastrointestinal injury: A focused update for clinicians. *J. Clin. Gastroenterol.* **50**, 5–10 (2016). 26524151 10.1097/MCG.0000000000000432

[7] Strate, L. L. & Gralnek, I. M. ACG clinical guideline: management of patients with acute lower Gastrointestinal bleeding. *Am. J. Gastroenterol.* **111**, 459–474 (2016). 26925883 10.1038/ajg.2016.41 PMC5099081

[8] Elenjickal, E. J., Travlos, C. K., Marques, P. & Mavrakanas, T. A. Anticoagulation in patients with chronic kidney disease. *Am. J. Nephrol.* **55**, 146–164 (2024). 38035566 10.1159/000535546 PMC10994631

[9] Januzzi, J. L. et al. The N-terminal Pro-BNP investigation of dyspnea in the emergency department (PRIDE) study. *Am. J. Cardiol.* **95**, 948–954 (2005). 15820160 10.1016/j.amjcard.2004.12.032

[10] Bytzer, P. et al. Prevalence of Gastrointestinal symptoms associated with diabetes mellitus: a population-based survey of 15,000 adults. *Arch. Intern. Med.* **161**, 1989–1996 (2001). 11525701 10.1001/archinte.161.16.1989

[11] Libby, P., Ridker, P. M. & Hansson, G. K. & Leducq transatlantic network on atherothrombosis. Inflammation in atherosclerosis: from pathophysiology to practice. *J. Am. Coll. Cardiol.* **54**, 2129–2138 (2009). 19942084 10.1016/j.jacc.2009.09.009 PMC2834169

[12] Lip, G. Y. H. & Halperin, J. L. Improving stroke risk stratification in atrial fibrillation. *Am. J. Med.* **123**, 484–488 (2010). 20569748 10.1016/j.amjmed.2009.12.013

[13] Rajkomar, A. et al. Scalable and accurate deep learning with electronic health records. *NPJ Digit. Med.* **1**, 18 (2018). 31304302 10.1038/s41746-018-0029-1 PMC6550175

[14] Krittanawong, C., Zhang, H., Wang, Z., Aydar, M. & Kitai, T. Artificial intelligence in precision cardiovascular medicine. *J. Am. Coll. Cardiol.* **69**, 2657–2664 (2017). 28545640 10.1016/j.jacc.2017.03.571

[15] Lundberg, S. M. et al. From local explanations to global Understanding with explainable AI for trees. *Nat. Mach. Intell.* **2**, 56–67 (2020). 32607472 10.1038/s42256-019-0138-9 PMC7326367

[16] Atsawarungruangkit, A., Habr, F. G. & RE Validation of a machine learning model that outperforms clinical risk scoring systems for upper Gastrointestinal bleeding. *Gastroenterology* **158**, 2307–2308 (2020). 32201179 10.1053/j.gastro.2020.02.061

[17] Weng, S. F., Reps, J., Kai, J., Garibaldi, J. M. & Qureshi, N. Can machine-learning improve cardiovascular risk prediction using routine clinical data? *PLoS One*. **12**, e0174944 (2017). 28376093 10.1371/journal.pone.0174944 PMC5380334

[18] Barkun, A. N. et al. Management of nonvariceal upper Gastrointestinal bleeding: guideline recommendations from the international consensus group. *Ann. Intern. Med.* **171**, 805–822 (2019). 31634917 10.7326/M19-1795 PMC7233308

[19] Gralnek, I. M. et al. Endoscopic diagnosis and management of nonvariceal upper Gastrointestinal hemorrhage (NVUGIH): European society of Gastrointestinal endoscopy (ESGE) Guideline - Update 2021. *Endoscopy* **53**, 300–332 (2021). 33567467 10.1055/a-1369-5274

[20] Liu, J. & Yin, T. [Application of artificial intelligence in precision cardiovascular medicine]. *Zhonghua Xin Xue Guan Bing Za Zhi*. **47**, 153–156 (2019). 30818944 10.3760/cma.j.issn.0253-3758.2019.02.013

[21] Shung, D. L. et al. Validation of a machine learning model that outperforms clinical risk scoring systems for upper Gastrointestinal bleeding. *Gastroenterology* **158**, 160–167 (2020). 31562847 10.1053/j.gastro.2019.09.009 PMC7004228

[22] Jordan, F. et al. Aspirin and other non-steroidal anti-inflammatory drugs for the prevention of dementia. *Cochrane Database Syst. Rev.* **4**, CD011459 (2020). 32352165 10.1002/14651858.CD011459.pub2 PMC7192366

[23] Ruff, C. T. et al. Comparison of the efficacy and safety of new oral anticoagulants with warfarin in patients with atrial fibrillation: a meta-analysis of randomised trials. *Lancet* **383**, 955–962 (2014). 24315724 10.1016/S0140-6736(13)62343-0

[24] Sandek, A. et al. Intestinal blood flow in patients with chronic heart failure: a link with bacterial growth, Gastrointestinal symptoms, and cachexia. *J. Am. Coll. Cardiol.* **64**, 1092–1102 (2014). 25212642 10.1016/j.jacc.2014.06.1179

[25] Moledina, D. G. & Perazella, M. A. PPIs and kidney disease: from AIN to CKD. *J. Nephrol.* **29**, 611–616 (2016). 27072818 10.1007/s40620-016-0309-2

[26] Sengupta, N. & Cifu, A. S. Management of patients with acute lower Gastrointestinal tract bleeding. *JAMA* **320**, 86–87 (2018). 29971385 10.1001/jama.2018.5684

[27] Illg, Z., Muller, G., Mueller, M., Nippert, J. & Allen, B. Analysis of absolute lymphocyte count in patients with COVID-19. *Am. J. Emerg. Med.* **46**, 16–19 (2021). 33706251 10.1016/j.ajem.2021.02.054 PMC7923864

[28] DeLoughery, T. G. Iron deficiency Anemia. *Med. Clin. North. Am.* **101**, 319–332 (2017). 28189173 10.1016/j.mcna.2016.09.004

[29] Tang, Y. D. & Katz, S. D. Anemia in chronic heart failure: prevalence, etiology, clinical correlates, and treatment options. *Circulation* **113**, 2454–2461 (2006). 16717164 10.1161/CIRCULATIONAHA.105.583666

[30] Kooiman, J. et al. Efficacy and safety of vitamin K-antagonists (VKA) for atrial fibrillation in non-dialysis dependent chronic kidney disease. *PLoS One*. **9**, e94420 (2014). 24817475 10.1371/journal.pone.0094420 PMC4015895

[31] Boccardo, P., Remuzzi, G. & Galbusera, M. Platelet dysfunction in renal failure. *Semin Thromb. Hemost.* **30**, 579–589 (2004). 15497100 10.1055/s-2004-835678

[32] Matzke, G. R. et al. Drug dosing consideration in patients with acute and chronic kidney disease-a clinical update from kidney disease: improving global outcomes (KDIGO). *Kidney Int.* **80**, 1122–1137 (2011). 21918498 10.1038/ki.2011.322

[33] Lip, G. Y. H. & Lane, D. A. Bleeding risk assessment in atrial fibrillation: observations on the use and misuse of bleeding risk scores. *J. Thromb. Haemost*. **14**, 1711–1714 (2016). 27296528 10.1111/jth.13386

[34] Danese, E., Montagnana, M., Cervellin, G., Lippi, G. & Hypercoagulability D-dimer and atrial fibrillation: an overview of biological and clinical evidence. *Ann. Med.* **46**, 364–371 (2014). 24863960 10.3109/07853890.2014.912835

[35] Agarwal, G. et al. Predictors and mortality risk of venous thromboembolism in patients with COVID-19: systematic review and meta-analysis of observational studies. *Ther. Adv. Cardiovasc. Dis.* **16**, 17539447221105013 (2022). 35762736 10.1177/17539447221105013 PMC9243575

[36] Yancy, C. W. et al. 2017 ACC/AHA/HFSA focused update of the 2013 ACCF/AHA guideline for the management of heart failure: A report of the American college of cardiology/american heart association task force on clinical practice guidelines and the heart failure society of America. *J. Am. Coll. Cardiol.* **70**, 776–803 (2017). 28461007 10.1016/j.jacc.2017.04.025

[37] Overland, M. K. & Dyspepsia *Med. Clin. North. Am.* **98**, 549–564 (2014). 24758960 10.1016/j.mcna.2014.01.007

[38] Parsons, P. E. et al. Lower tidal volume ventilation and plasma cytokine markers of inflammation in patients with acute lung injury. *Crit Care Med* 33, 1–6; discussion 230–232 (2005). 10.1097/01.ccm.0000149854.61192.dc 15644641

[39] Lanza, F. L., Chan, F. K. L. & Quigley, E. M. M. & practice parameters committee of the American college of gastroenterology. Guidelines for prevention of NSAID-related ulcer complications. *Am. J. Gastroenterol.* **104**, 728–738 (2009). 19240698 10.1038/ajg.2009.115

[40] Cryer, B. & Mahaffey, K. W. Gastrointestinal ulcers, role of aspirin, and clinical outcomes: pathobiology, diagnosis, and treatment. *J. Multidiscip Healthc.* **7**, 137–146 (2014). 24741318 10.2147/JMDH.S54324 PMC3970722

[41] Lundberg, S. M. et al. Explainable machine-learning predictions for the prevention of hypoxaemia during surgery. *Nat. Biomed. Eng.* **2**, 749–760 (2018). 31001455 10.1038/s41551-018-0304-0 PMC6467492

[42] Tomašev, N. et al. A clinically applicable approach to continuous prediction of future acute kidney injury. *Nature* **572**, 116–119 (2019). 31367026 10.1038/s41586-019-1390-1 PMC6722431

[43] Goldstein, B. A., Navar, A. M., Pencina, M. J. & Ioannidis, J. P. A. Opportunities and challenges in developing risk prediction models with electronic health records data: a systematic review. *J. Am. Med. Inf. Assoc.* **24**, 198–208 (2017). 10.1093/jamia/ocw042 PMC5201180 27189013

[44] Moons, K. G. M. et al. Transparent reporting of a multivariable prediction model for individual prognosis or diagnosis (TRIPOD): explanation and elaboration. *Ann. Intern. Med.* **162**, W1–73 (2015). 25560730 10.7326/M14-0698

[45] Rudin, C. Stop explaining black box machine learning models for high stakes decisions and use interpretable models instead. *Nat. Mach. Intell.* **1**, 206–215 (2019). 35603010 10.1038/s42256-019-0048-x PMC9122117

