# Supplementary material for: Prediction of gastrointestinal hemorrhage in cardiology inpatients using an interpretable XGBoost model
Source: Sci Rep. 2025 Jul 12;15:25240. doi: 10.1038/s41598-025-10906-1 (PMC12255804; doi:10.1038/s41598-025-10906-1)
Supplement: Supplementary file 1 — Supplementary Material 1 [file 41598_2025_10906_MOESM1_ESM.docx]

**Supplementary Table 1** Mean DeLong Z-values for the training set.

| Name | XGBClassifier | LogisticRegression | LGBMClassifier | RandomForestClassifier | GradientBoostingClassifier | GaussianNB | KNeighborsClassifier |
| --- | --- | --- | --- | --- | --- | --- | --- |
| XGBClassifier | NA | 7.263 | 1.055 | 2.283 | 4.913 | 8.968 | 4.393 |
| LogisticRegression | 7.263 | NA | 7.162 | 6.31 | 6.153 | 5.64 | 3.283 |
| LGBMClassifier | 1.055 | 7.162 | NA | 2.344 | 5.052 | 8.953 | 4.115 |
| RandomForestClassifier | 2.283 | 6.31 | 2.344 | NA | 3.852 | 8.36 | 3.046 |
| GradientBoostingClassifier | 4.913 | 6.153 | 5.052 | 3.852 | NA | 8.509 | 0.935 |
| GaussianNB | 8.968 | 5.64 | 8.953 | 8.36 | 8.509 | NA | 5.584 |
| KNeighborsClassifier | 4.393 | 3.283 | 4.115 | 3.046 | 0.935 | 5.584 | NA |

**Supplementary Table 2** Mean DeLong Z-values for the validation set.

| Name | XGBClassifier | LogisticRegression | LGBMClassifier | RandomForestClassifier | GradientBoostingClassifier | GaussianNB | KNeighborsClassifier |
| --- | --- | --- | --- | --- | --- | --- | --- |
| XGBClassifier | NA | 0.0 | 0.379 | 0.035 | 0.0 | 0.0 | 0.0 |
| LogisticRegression | 0.0 | NA | 0.0 | 0.0 | 0.0 | 0.0 | 0.005 |
| LGBMClassifier | 0.379 | 0.0 | NA | 0.052 | 0.0 | 0.0 | 0.0 |
| RandomForestClassifier | 0.035 | 0.0 | 0.052 | NA | 0.023 | 0.0 | 0.024 |
| GradientBoostingClassifier | 0.0 | 0.0 | 0.0 | 0.023 | NA | 0.0 | 0.43 |
| GaussianNB | 0.0 | 0.0 | 0.0 | 0.0 | 0.0 | NA | 0.0 |
| KNeighborsClassifier | 0.0 | 0.005 | 0.0 | 0.024 | 0.43 | 0.0 | NA |

**Supplementary Table 3** Performance metrics of the AdaBoost and Decision Tree models on the independent test set.

| Model | AUC | Cutoff | Accuracy | Sensitivity | Specificity | PPV | NPV | F1 Score |
| --- | --- | --- | --- | --- | --- | --- | --- | --- |
| XGBoost | 0.995 | 0.816 | 0.974 | 0.790 | 0.991 | 0.895 | 0.981 | 0.839 |
| AdaBoost | 0.962 | 0.488 | 0.9 | 0.896 | 0.901 | 0.513 | 0.987 | 0.652 |
| DecisionTree | 0.896 | 0.828 | 0.959 | 0.819 | 0.973 | 0.755 | 0.981 | 0.786 |
